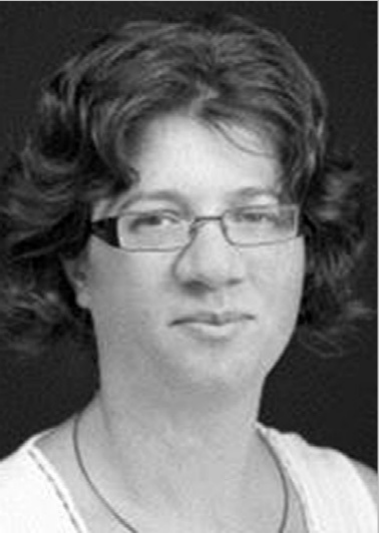# Sustainable Separations of C_4_‐Hydrocarbons by Using Microporous Materials

**DOI:** 10.1002/cssc.201700657

**Published:** 2017-09-18

**Authors:** Mascha Gehre, Zhiyong Guo, Gadi Rothenberg, Stefania Tanase

**Affiliations:** ^1^ Van ‘t Hoff Institute for Molecular Sciences University of Amsterdam Science Park 904 1098 XH Amsterdam The Netherlands; ^2^ College of Materials Science and Engineering Fuzhou University Fuzhou Fujian 350108 P. R. China

**Keywords:** adsorption, hydrocarbons, microporous materials, metal–organic frameworks, separation

## Abstract

Petrochemical refineries must separate hydrocarbon mixtures on a large scale for the production of fuels and chemicals. Typically, these hydrocarbons are separated by distillation, which is extremely energy intensive. This high energy cost can be mitigated by developing materials that can enable efficient adsorptive separation. In this critical review, the principles of adsorptive separation are outlined, and then the case for C_4_ separations by using zeolites and metal–organic frameworks (MOFs) is examined. By analyzing both experimental and theoretical studies, the challenges and opportunities in C_4_ separation are outlined, with a focus on the separation mechanisms and structure–selectivity correlations. Zeolites are commonly used as adsorbents and, in some cases, can separate C_4_ mixtures well. The pore sizes of eight‐membered‐ring zeolites, for example, are in the order of the kinetic diameters of C_4_ isomers. Although zeolites have the advantage of a rigid and highly stable structure, this is often difficult to functionalize. MOFs are attractive candidates for hydrocarbon separation because their pores can be tailored to optimize the adsorbate–adsorbent interactions. MOF‐5 and ZIF‐7 show promising results in separating all C_4_ isomers, but breakthrough experiments under industrial conditions are needed to confirm these results. Moreover, the flexibility of the MOF structures could hamper their application under industrial conditions. Adsorptive separation is a promising viable alternative and it is likely to play an increasingly important role in tomorrow's refineries.

## 1 Introduction

Valorization of C_4_ hydrocarbons (butane, 1‐butene, 2‐butene, isobutene, and 1,3‐butadiene) is an important research topic because of the market size and versatility of these bulk chemicals.^1^ Compared with ethene and propene, the main challenge in C_4_ compounds is their upgrading to high‐value end products.^2^ Large amounts of C_4_ hydrocarbons are coproduced with ethylene in steam cracking, as well as alongside gasoline in the fluid catalytic cracking (FCC) process.^3^ Moreover, there is increasing interest in valorizing the C_4_ hydrocarbons that arise from coal liquefaction and biomass refining. This is especially important in China, where coal is the main carbon resource.^4^, ^5^ Typical C_4_ streams from FCC, steam cracking, and the methanol‐to‐olefins (MTO) processes contain mainly 1‐butene, 2‐butene, isobutene, and 1,3‐butadiene (see Table 1). The actual ratios vary, due to different compositions of the raw materials.^2^

**Table 1:** Composition of C_4_ streams obtained from FCC, MTO, and steam cracking.^2^, ^5^

Compound	Mass fraction [%]		
	FCC	MTO	steam cracking
isobutane	35–45	0.2	0–2
*n*‐butane	7–42	4	2–5
isobutene	10–20	2–4	18–32
1‐butene	9–12	20–26	14–22
2‐butene	20–29.5	65–70	5–15
butadiene	0–0.5	–	35–50

**Scheme 1:**
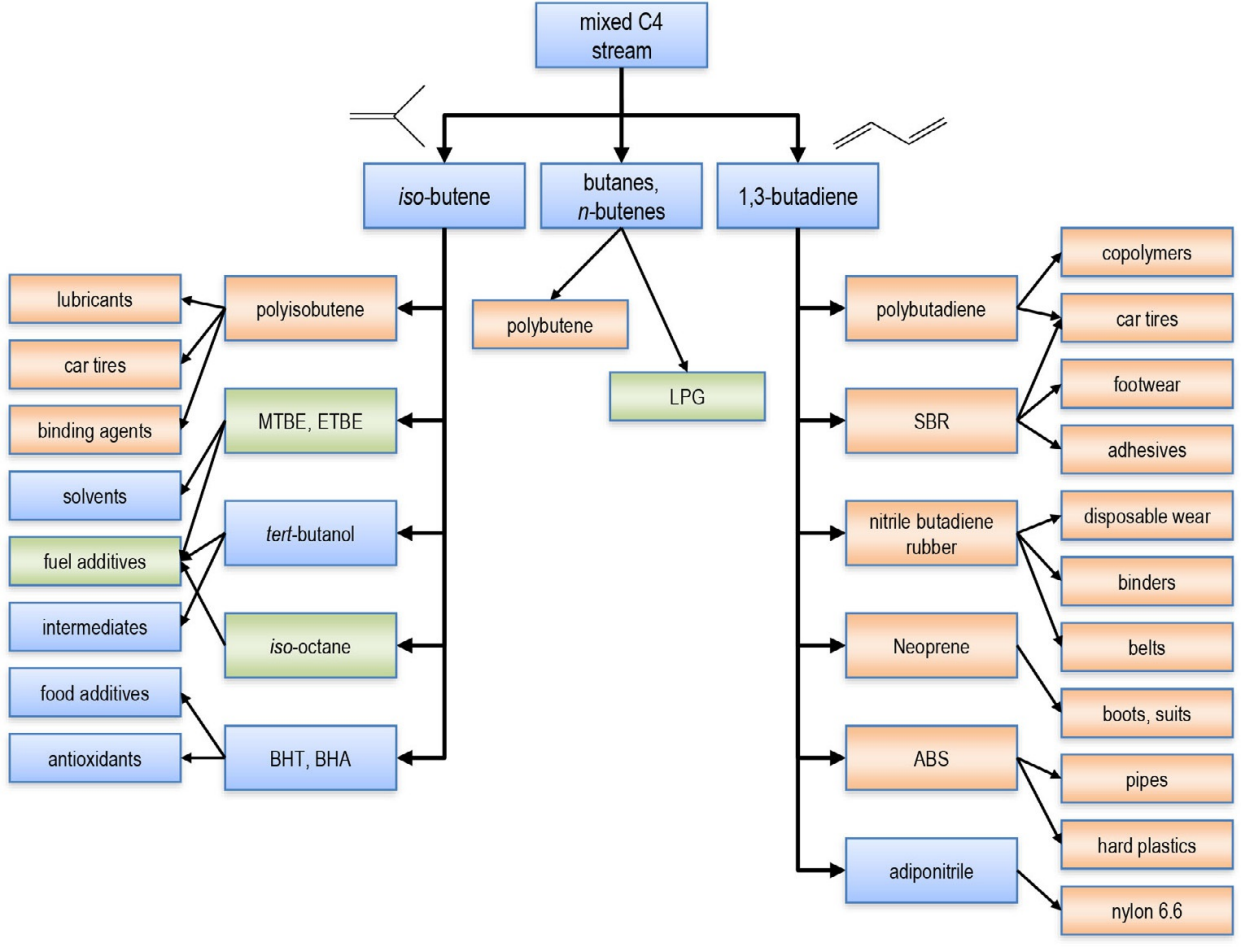
Tree view of the industrial applications of C_4_ streams. Polymers and polymer applications are highlighted in orange; fuels and fuel additives are highlighted in green. LPG=liquefied petroleum gas; MTBE=methyl‐*tert*‐butyl ether; ETBE=ethyl‐*tert*‐butyl ether; BHT=butylated hydroxytoluene; BHA= butylated hydroxyanisole; SBR=styrene butadiene rubber.

Currently, only 1,3‐butadiene, isobutene, and 1‐butene are on the market as intermediates with standardized product purities.^2^ 1,3‐Butadiene is mainly used as a monomer in the manufacture of synthetic rubbers and elastomers.^2^ It is also used as a monomer for styrene–butadiene (S/B) latex, acrylonitrile–butadiene–styrene (ABS) resins, and high‐impact polystyrene (HIPS).^2^ Gaseous isobutene is another important petrochemical building block. About 15 million tons per year of isobutene are derived from oil and converted into fuels, plastics, and elastomers.^6^ The demand for *n*‐butenes is also high because of the large markets for alkylate gasoline, detergent alcohols, synthetic lubricants, and plasticizers.^6^ For most of these applications, and especially for polymerization, the purity of the C_4_ components is critical. Scheme 1 shows the valorization chain of C_4_ hydrocarbons.

In large‐scale chemical processes, separation and purification accounts for much of the costs (both capital expenditure (CapEx) and operating expenditure (OpEx)). This is mainly due to the high energy demand of these unit operations. In most of these processes, it is the separation units that incur most of the environmental burden in terms of CO_2_ footprint and energy costs.

Unfortunately, the physical properties of C_4_ isomers are similar (see Table 2). The boiling points of 1‐butene (266.92 K) and isobutene (266.25 K) are practically identical. Separating such compounds by distillation is extremely costly.^2^, ^7^ 1,3‐Butadiene is usually separated by extractive distillation with acetonitrile and *N*,*N*‐dimethylformamide.^8^ Isobutene is removed under mild acid catalysis upon which it forms selectively MTBE or *tert*‐butanol.^9^ The challenging step is the separation of 1‐butene from the isomers of 2‐butene. High‐purity 1‐butene is crucial in the production of linear low‐density polyethylene (LLDP).^10^ Isobutene is usually absorbed by using molecular sieves.^11^ The isomers of 2‐butene are not further separated because they react analogously in further processing by dehydrogenation, oligomerization, or alkylation.^3^ A possible separation could, however, lead to new applications, including the production of high‐performance polymers.^4^ Hence, developing more sustainable processes for C_4_ separation can create substantial value for these large markets.

**Table 2:** Physical properties of C_4_ hydrocarbons.^14^, ^18^, ^43^

Compound	B.p. [K]	Kinetic diameter [Å]	Polarizability [10^−25^ cm^3^]	Dipole moment^[a]^ [×10^18^ esu cm]
butane	272.66	4.687	82	0.05
isobutane	261.34	5.278	81.4–82.9	0.132
1‐butene	266.92	4.46	81	0.359–0.438
*cis*‐2‐butene	276.87	4.94	82	0.30
*trans*‐2‐butene	274.03	4.31	81.82	0.00
1,3‐butadiene	268.62	4.31	86.4	0.00
isobutene	266.25	4.840	80	0.50

[a] 1 Debye=10^−18^ esu cm.

Separation technologies based on adsorption by using microporous materials are alternative energy‐efficient purification methods.^12^ Microporous materials can separate compounds by using their physical properties, such as kinetic diameter, polarizability, acid–base nature, coordinative properties, permanent dipole moment, and quadrupole moment.^13^ This can give advantages in terms of product recovery and purity, as well as energy costs.^7^, ^14^, ^15^ Herein, we look at the state of the art of C_4_ separation by using microporous materials, with recommendations for possible industrial applications

## 2 Zeolites and MOFs: Two Classes of Microporous Materials

High‐surface‐area microporous materials (materials with pore diameters <2 nm) are the subject of continued research.^16^ Much of this research focuses on zeolites^17^, ^18^, ^19^ and metal– organic frameworks (MOFs).^7^, ^16^, ^20^, ^21^ These two families of microporous materials are highly relevant for C_4_ separations.

Zeolites are microporous aluminosilicates with a well‐defined crystalline structure. All zeolites have rigid skeletons. They are highly porous and act as molecular sieves.^22^ Because their pore sizes are in the same order of magnitude as the sizes of small gas molecules, some zeolites are attractive for molecular separation, for example, as packed beds or microporous membranes.^22^ Today, the term zeolite has broadened to include all microporous silica‐based solids with crystalline walls. Consequently, it also includes materials in which some of the silicon ions are substituted by other elements.^18^ The silicon/aluminum ratios may vary to give hydrophilic and ‐phobic zeolite structures. This is because aluminum ions induce an overall negative charge in the zeolite framework, which is counterbalanced by nonframework cations. These, in turn, may interact with specific adsorbate molecules. The result is a higher selectivity of aluminum‐rich zeolites towards certain molecules.^23^, ^24^, ^25^

MOFs are a relatively new class of microporous materials.^17^ They are built from metal ions (or clusters of metal ions) linked by organic ligands.^16^ MOFs have an advantage over zeolites because chemical modification of the organic linkers can provide tailored materials for specific applications. For example, the length of the organic linker often defines the pore size of a given material.^26^ Currently, there are about 10 000 experimentally known MOFs (versus <300 zeolite types).^16^ The main disadvantage of MOFs is their low thermal stability (typically stable to 350–400 °C, rarely 500 °C). This rules out high‐temperature processes, but MOFs can be used for gas storage, separation, and purification.^27^ Unlike zeolites, which are always rigid, MOFs can be flexible, responding dynamically to guest molecules or to external stimuli, such as pressure and temperature.^13^ With their exceptionally high porosity and relative simple self‐assembly, MOFs are interesting for both fundamental studies and practical applications.^12^

Rigid MOFs have permanent porosity and well‐defined pores or channels, similar to zeolites;^16^ this makes them good molecular sieves.^28^ Pore size is usually the dominating parameter in separating small molecules. The molecular kinetic diameter is a key factor for separation efficiency. For larger molecules, both the size and nature of the pores (hydrophilic/‐phobic, aliphatic/aromatic) are important.^13^ Flexible MOFs have an added advantage. For example, in gate‐opening transitions, a non‐porous material can become an open, microporous one.^29^ Similarly, “breathing” occurs when the pores expand (or contract) reversibly.^30^ In both cases, changing the framework structure will change the adsorption capacity.^31^ Combining this flexibility with a functional surface can increase the selectivity of gas separation processes.^2^ Lewis acidic MOFs have unsaturated metal centers that act as Lewis acid sites. Similar to aluminum‐rich zeolites, these MOFs interact with certain sorbate molecules through specific interactions. This makes them attractive for separating molecules with coordinative active groups.^13^

## 3 Adsorptive Separation

Adsorptive separation is a sustainable process that is widely used by the chemical industry.^11^ The process implies the separation of a molecular mixture based on differences in adsorption–desorption behavior of the distinct components in the mixture. In such a process, the mixture is first contacted with an adsorbent material under specific conditions to allow the selective removal of one or more components.^32^ For gas‐phase separations, the regeneration of the adsorbent is usually achieved by changing the pressure or temperature of the system, such processes are known as pressure swing adsorption (PSA) or temperature swing adsorption (TSA), respectively. The main advantage of such processes is that they can be operated at low adsorbent loading because the selectivity between components in the gas phase is greatest in the Henry's law region. For liquid‐phase separations, a desorbent is required that displaces the adsorbed species preferentially from the adsorbent. Economic viability of both gas‐ and liquid‐phase adsorptive separations requires adsorbent materials that facilitate high separation selectivity, high adsorption capacity, and short duration cycles.^32^

Adsorptive separation by using porous materials is a combination of steric, equilibrium, and kinetic separations.^12^ The size of the adsorptive molecules limits the range of pore and/or window accessibility. Generally, a smaller pore results in stronger interaction with the adsorbent. However, if the pores are too narrow (relative to the size of the adsorbates), repulsive forces increase and the interaction weakens.^17^ Steric separation prevents certain components of a mixture from entering the pores. Such size/shape exclusion is common in zeolites and rigid MOFs. Here, both the cross‐sectional size and shape of the adsorbate affect the selective adsorption. The former is known as the kinetic diameter or collision diameter; this is the intermolecular distance of the closest approach for molecules colliding with zero initial kinetic energy.

If the pores are large enough for all components of a mixture to pass, preferential adsorption can occur. This is known as the thermodynamic equilibrium effect.^12^ The strength of the interaction depends on the surface of the adsorbent and properties of the adsorbate. These are polarizability, magnetic susceptibility, acid–base nature, coordinative properties, permanent dipole moment, and quadrupole moment.^12^, ^13^ Kinetic separation, also known as partial molecular sieve action, is an alternative when equilibrium separation is not feasible. Although the amounts of different components of a mixture adsorbed at equilibrium are similar, some components may diffuse faster than others. The different diffusing rates may be used to separate the components. For kinetic separation, the pore diameter of the adsorbent needs to be between the kinetic diameters of the two molecules to be separated.^12^

The adsorption quantity of a component at a given temperature is measured by an adsorption isotherm.^12^ This isotherm relates the amount of substance adsorbed at equilibrium to the pressure of the adsorptive in the mixture phase.^12^, ^13^ For flexible MOFs, the adsorption isotherms cannot be classified according to the IUPAC scheme because such MOFs can undergo structural changes when guest molecules enter.^13^ Consequently, the isotherms show distinct steps and hysteresis in the adsorption and desorption phases.^33^

Adsorption is an exothermic process, whereas desorption is endothermic. Thus, the temperature changes within the adsorbent during adsorption/desorption. This temperature is a key variable in determining local adsorption equilibria and ultimately governs the separation performance of the material.^34^ The isothermic heat of adsorption determines the variation range of the temperature change that takes place during adsorption processes. High isosteric adsorption heats imply a strong interaction between guest molecules and the host. Therefore, the strength of the interaction needs to be optimized to reach high adsorption capacities.^2^

Most model studies on mixture separations rely on single‐component isotherms. Yet, in reality, pore blocking and cooperative effects between different components play a key role. Breakthrough curves of multicomponent mixtures give valuable information about the separation efficiency of a material towards a gas mixture. These curves are measured by flowing the mixture through a thermostated bed of adsorbent and monitoring the effluent (by GC or MS, for example). Ideally, the adsorbate to be removed should be strongly adsorbed and not be detected in the effluent until saturation. Once saturation is reached, the adsorbent is regenerated.^13^

## 4 Separating C_4_ Hydrocarbons with Zeolites

The separation of C_4_ hydrocarbons is reported on several zeolite structures.^35^, ^36^ The pore size of these materials range from approximately 2 nm down to the order of the kinetic diameters of the C_4_ isomers. We discuss the performance of various zeolite structures in terms of their pore size. First, we analyze the Faujasites (FAUs; which have the largest pores), then medium‐pore‐sized 10‐membered‐ring (10MR) MFI‐type zeolites, and finally 8‐membered‐ring (8MR) zeolites.

**Figure 1:**
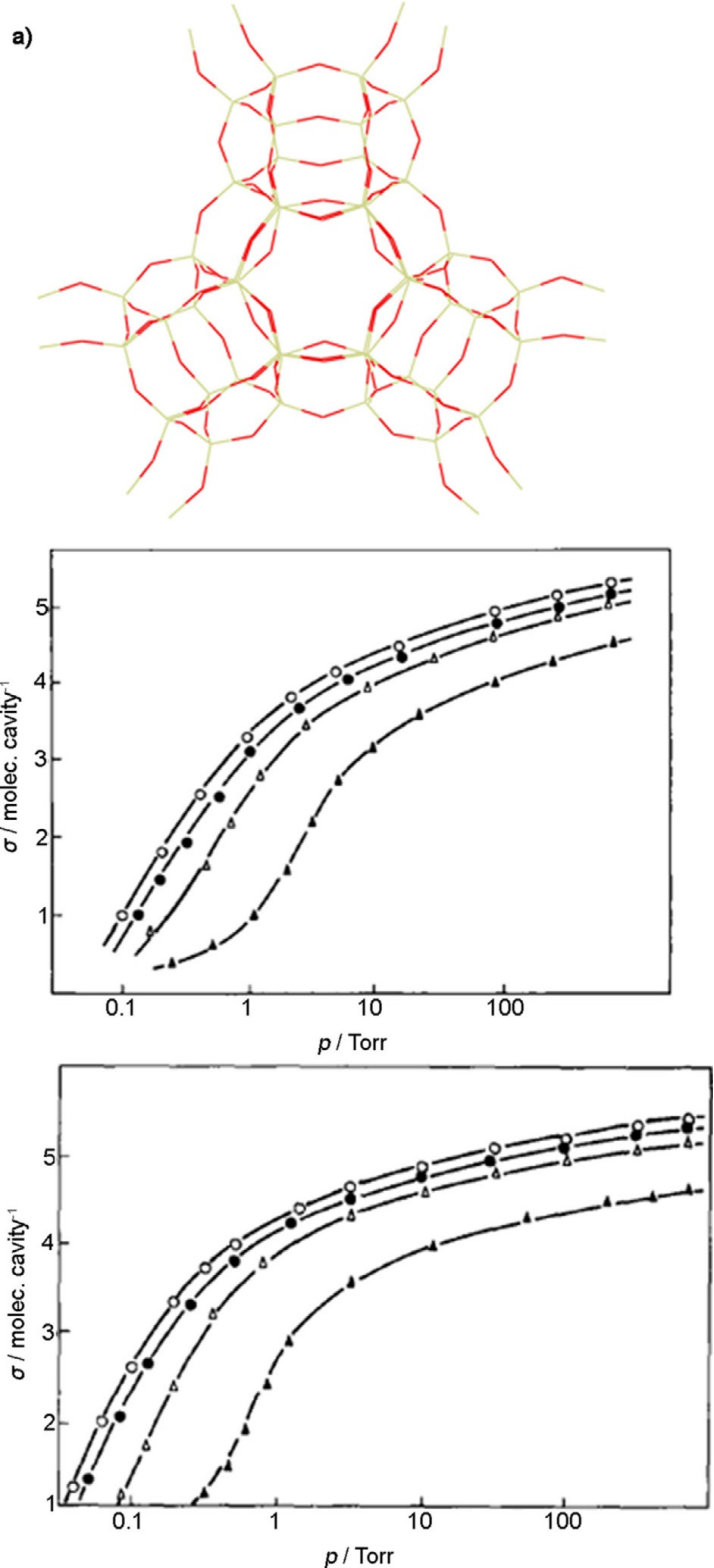
a) Framework structure of FAU. b) Adsorption isotherms and c) heat of adsorption of C_4_ hydrocarbons on zeolite X at 303 K for *cis*‐2‐butene (○),1‐butene (•), *trans*‐2‐butene (Δ), and butane (▴).^38^ 1 torr=133.322 Pa. Parts (a) and (c) reproduced with permission from Elsevier.

FAU zeolites consist of sodalite cages interconnected in such a way that 15 Å diameter supercages are accessible through 7.4 Å diameter windows in a tetrahedral arrangement (Figure 1 a).^25^ Their composition is Na_*x*_Al_*x*_Si_192−*x*_O_384_ (0≤*x*≤96). These zeolites exist as high‐silicate zeolite X or as high‐aluminate zeolite Y.^24^ The former contains between 77 and 96 aluminum ions per unit cell, whereas the latter has <77 aluminum ions per unit cell. Because aluminum ions induce the presence of negative charges in the framework (which are then counterbalanced by nonframework metal cations), the hydrophilicity of FAU zeolites increases as the silicon/aluminum ratio decreases.^24^, ^25^, ^38^ We refer to zeolite X as M‐X and to zeolite Y as M‐Y, in which M denotes the nonframework metal cations. Although the pores of FAU zeolites are much larger than the kinetic diameters of the C_4_ isomers (see Table 2), they can still separate these isomers by using differences in the dipole moments or electric polarizabilities.

### 4.1 *n*‐Butane

Harlfinger et al. studied the C_4_ single‐component adsorption isotherms and integral heats of adsorption on Na‐X (see structure in Figure 1 a).^38^ This zeolite has a composition of Na_68_Al_68_Si_124_O_260,_ with a Si/Al ratio of about 1.8. Figure 1 b and c shows the isotherms at 303 K and the heat of adsorption of C_4_ hydrocarbons. The adsorption order is *cis*‐2‐butene> 1‐butene>*trans*‐2‐butene>butane.^38^ This order reflects differences in dipole moments, electric polarizabilities, and molecular geometries. *cis*‐2‐Butene adsorbs preferentially owing to the arrangement of methyl groups to one side of the molecule and to the magnitude and direction of its dipole moment. 1‐Butene has a weaker interaction with the zeolite than that of *cis*‐2‐butene because it has a terminal double bond. Indeed, *trans*‐2‐butene shows the weakest interaction because it has no dipole moment and its double bond is sterically hindered.^38^ The difference between the isothermal behavior of the isomeric butenes and that of butane is related to the absence of a double bond in the latter.^37^ The same study also determined that the adsorption order of *cis*‐2‐butene, 1‐butene, *trans*‐2‐butene, and butane remained unchanged for alkali modifications of the zeolite. Harlfinger et al. also determined that, for alkali modifications of the zeolite, the adsorption order of *cis*‐2‐butene, 1‐butene, *trans*‐2‐butene, and butane remained unchanged.^38^ It was expected that exchanging Na^+^ with smaller (Li^+^) or larger (K^+^, Rb^+^, and Cs^+^) cations would result in a change in the electric field strength in the interior of the zeolite and, hence, in a change in heterogeneity of the zeolite surface. Because of the larger charge‐to‐radius ratio, greater interactions can be expected for Li^+^ than those for Na^+^. Accordingly, these interactions should become smaller for K^+^, Rb^+^, and Cs^+^. These studies demonstrated that *cis*‐2‐butene, irrespective of the type of cation involved and because of its specific geometry and dipole moment, had the most favorable arrangement in the large cavity of the zeolite, compared with both 1‐butene and *trans*‐2‐butene.^38^

**Figure 2:**
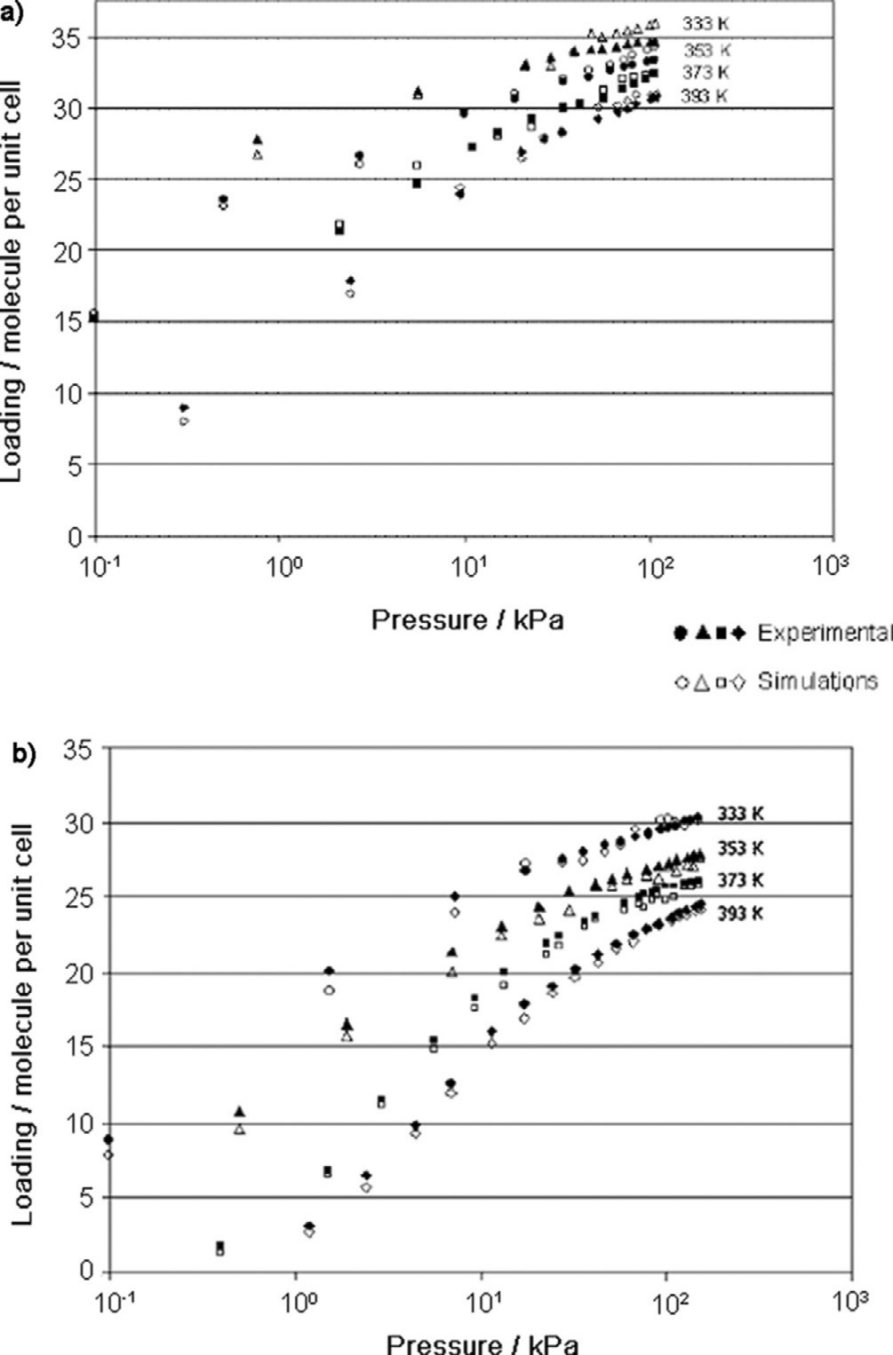
Comparison between simulations performed by Granato et al.^23^ (open symbols) and experimental data of Lamia et al.^24^ (closed symbols) for 1‐butene adsorption isotherms (a) and isobutane adsorption isotherms (b) on zeolite 13X. Reproduced with permission from The American Chemical Society.

Lamia et al. determined the single‐component adsorption isotherms of isobutane and 1‐butene on Na‐13X.^24^, ^39^, ^40^ This zeolite has the composition Na_88_Al_88_Si_104_O_384_ and a lower Si/Al ratio than that of the structure reported by Harlfinger et al.^38^ Figure 2 shows that the extracted saturation capacity for 1‐butene is higher than that of isobutene on Na‐13X. This is due to the kinetic diameter of 1‐butene (4.83 Å), which is smaller than that of isobutane (5.28 Å). Although both molecules can access the supercages of the zeolite through the 7.4 Å diameter windows, the number of isobutane molecules per cage is lower. Figure 2 also shows the simulated equilibrium adsorption isotherms of isobutene and 1‐butene in Na‐13X, as determined by Granato et al.^23^ We see that the determined set of Lennard–Jones parameters successfully reproduces the equilibrium adsorption properties of 1‐butene and isobutene. In principle, the proposed extended force field can be used to predict the adsorption properties of mixtures of C_4_ isomers on zeolite 13X.^24^ The differences between the single‐component adsorption isotherms of the C_4_ isomers reported by Harlfinger et al.^38^ and Lamia et al.^39^ are small.

Tielens et al. showed that the adsorption capacity of Na‐Y zeolites was even lower than that of Na‐X zeolites.^22^ They measured the heats of adsorption of 1‐butene, *cis*‐2‐butene, *trans*‐2‐butene, and isobutene on Na‐Y with a Si/Al ratio of 3.8. Table 3 lists the Henry constants, adsorption enthalpies, and adsorption entropies of the isomers, as well as the adsorption enthalpies reported by Tielens et al.^22^ The Henry constants decrease in the order isobutene>*cis*‐2‐butene>1‐butene>*trans*‐2‐butene>butane. This trend was also reported by Harlfinger et al.,^38^ albeit with lower values due to using a zeolite with a lower aluminum content (and thus, fewer interaction sites).

**Table 3:** Experimental Henry constants (*K*′), adsorption enthalpies, and adsorption entropies of the butene isomers on zeolite Y.^22^

Compound	Experimental			Calculated
	*K*′ [mol kg^−1^ Pa^−1^] (170 °C)	ΔH⊖0 [kJ mol^−1^]	ΔS⊖0 [kJ mol^−1^]	ΔH⊖0 [kJ mol^−1^]^[a]^
isobutene	1.41×10^−4^	−49.0±0.6	−80.0	
1‐butene	1.15×10^−4^	−48.9±0.3	−81.2	−60.5
*cis*‐2‐butene	1.26×10^−4^	−49.0±0.2	−80.9	−60.8
*trans*‐2‐butene	1.01×10^−4^	−48.7±0.4	−81.9	−59.0
*n*‐butene	3.19×10^−5^	−37.1±0.1	−67.3	−51.5

[a] Results from Ref. 38.

**Figure 3:**
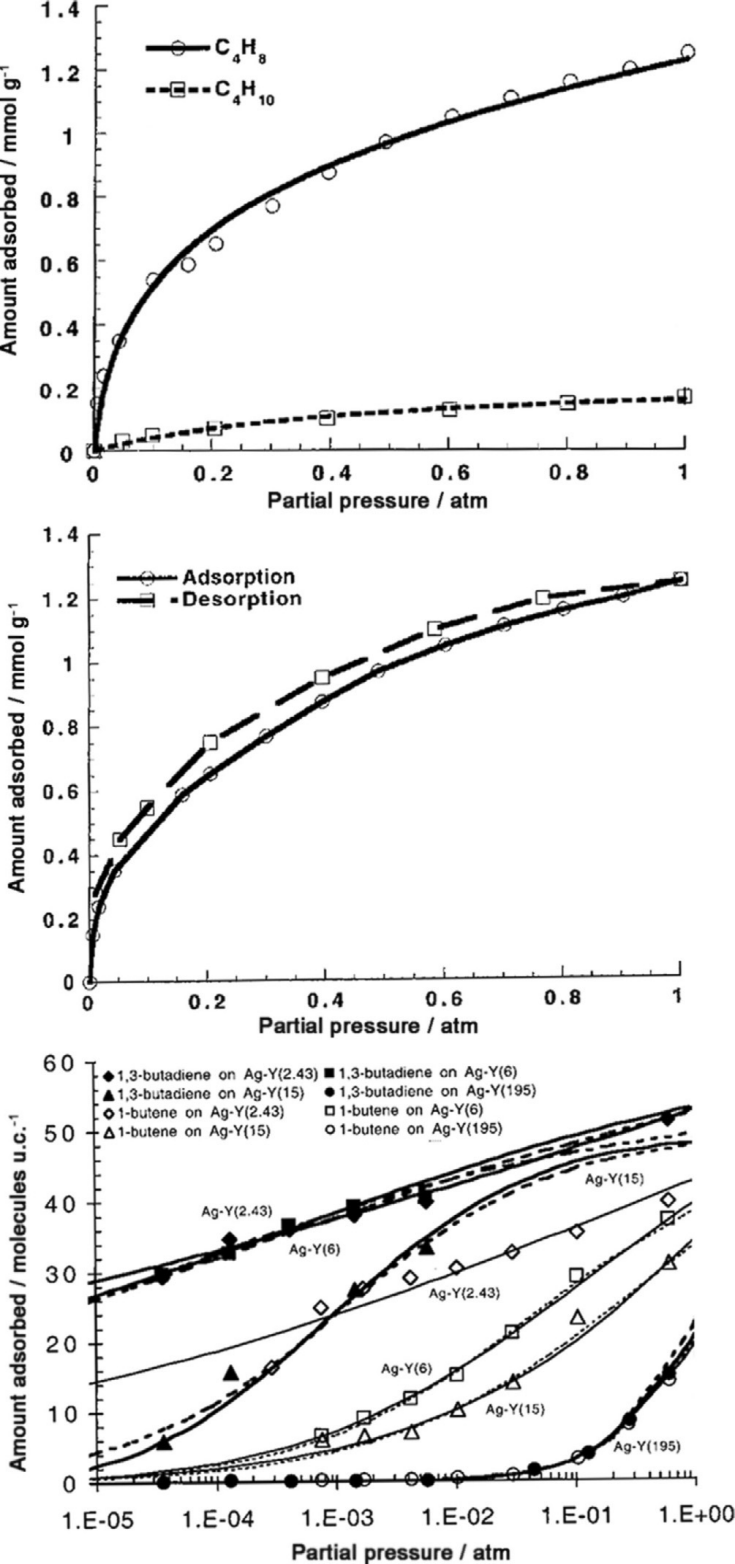
Equilibrium isotherms of a) 1‐butene (C_4_H_8_) and butane (C_4_H_10_), and b) 1,3‐butadiene (C_4_H_6_) and butane at 343 K on Ag‐Y.^44^ c) Pure component equilibrium isotherms at 393 K for 1,3‐butadiene and 1‐butene on Ag‐Y with different Si/Al ratios. Ag‐Y(2.43) refers to a silver‐ion‐exchanged Y‐type zeolite with a Si/Al ratio of 2.34.^45^ 1 atm=101325 Pa. Reproduced with permission from The American Chemical Society.

The examples discussed above exclude the 1,3‐butadiene isomer because in 1976 Priegnitz patented a process for separating 1,3‐butadiene by selective adsorption on a zeolite X adsorbent containing sodium or potassium as nonframework cations.^41^ However, the adsorption capacity of the zeolite at low diene partial pressures cannot satisfy today's purity requirements.^42^ Studies with zeolite Y were performed, in which 100 % of the Na^+^ nonframework cations were exchanged for transition‐metal ions. These ions can participate in both σ bonding to carbon and π complexation.^43^ Thanks to the π bonds, one may achieve high selectivity and high capacity for the separation of C_4_‐butene isomers on transition‐metalmodified zeolites. These bonds are weak enough to be broken by simply raising the temperature or decreasing the pressure.^44^ This was the first application of π‐complexation adsorbents in the separation of C_4_ hydrocarbons, and showed that Ag‐Y was more selective for 1‐butene and 1,3‐butadiene over butane (see Figure 3 a,b).

Studying the influence of the Ag^+^ ion content in Ag‐Y on 1,3‐butadiene and 1‐butene adsorption shows that the adsorption decreases when the Si/Al ratio increases.^45^ This is because fewer Ag^+^ cations are available in the zeolites with a higher Si content (Figure 3 c). The adsorption of 1,3‐butandiene on Ag^+^–Na^+^ mixed ion‐exchanged zeolites (AgNa‐Y) was also studied. At 1 bar (=10^5^ Pa), adsorption of 1,3‐butadiene and 1‐butene was still comparable to that of Ag‐Y when 70 % of the Ag^+^ ions were exchanged with Na^+^. The problem is that C_4_ streams contain traces of H_2_S, C_2_H_2_, and H_2_; all of which can poison Ag^+^ ions. Therefore, a Cu‐Y zeolite was used instead of Ag‐Y and its stability under H_2_ and H_2_S exposure was studied.^46^ The results showed that exposure to H_2_S and H_2_ had no effect on the 1,3‐butadiene uptake. Furthermore, H_2_ exposure had no effect on 1‐butene uptake, whereas H_2_S exposure slightly decreased the 1‐butene uptake on Cu‐Y (because H_2_S adsorbs irreversibly on the framework). Notably, in all experiments, the 1,3‐butadiene and 1‐butene uptakes on Cu‐Y were higher than that on Ag‐Y because of the higher pore volume of Cu‐Y. Although these results are promising, there are no reports of mixed‐gas breakthrough experiments on Cu‐Y.

**Figure 4:**
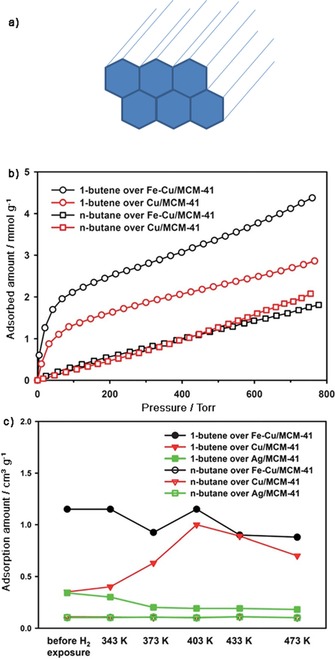
a) The non‐interconnected pore system of MCM‐41 constructed of channels with a diameter of 28 Å.^47^, ^48^ b) Adsorption isotherms of 1‐butene and *n*‐butane over Fe‐Cu/MCM‐41 and Cu/MCM‐41 adsorbents, obtained at 313 K. c) Adsorption amounts of 1‐butene and *n*‐butane when a 0.26 % binary mixture of the two C_4_ isomers in helium flows at a rate of 8 cm^3^ min^−1^ through Ag/MCM‐41, Cu/MCM‐41, and Fe‐Cu/MCM‐41.^48^ Parts (b) and (c) reproduced with permission from The American Chemical Society.

One mesoporous material that gives additional insight into copper‐based adsorbents is Cu‐Fe/MCM‐41, which contains ferrous/cuprous (Fe^2+^/Cu^+^) ions. MCM‐41 has separate channel pores with a diameter of 28 Å (Figure 4 a).^47^, ^48^ Single‐component adsorption isotherms (Figure 4 b) and breakthrough experiments (Figure 4 c) both showed that Fe^2+^ ion species stabilized the Cu^+^ species in the framework, increasing 1‐butene/butane separation. Indeed, Fe^2+^ species may prevent oxidation and reduction of Cu^+^ species during adsorbent preparation, the separation process, and in the presence of H_2_.^48^ In the Cu‐Y framework, the Cu^+^/Cu^2+^ ratio was 0.5 and incorporating Fe^2+^ ions into the zeolite also improved the separation of 1‐butene from butane.

**Figure 5:**
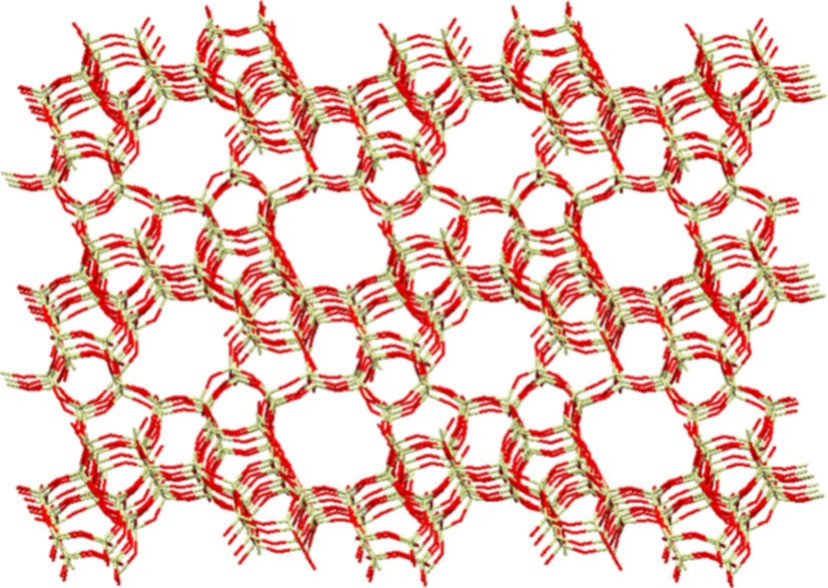
Molecular structure of MFI zeolite, showing well‐defined pores and channels in the zeolite.

The MFI‐type zeolites silicate‐1 and ZSM‐5 are among the most studied and most widely used zeolites.^49^ Figure 5 shows a drawing of their zigzag channels along the *x* direction that are intersected by straight channels along the *y* direction. Both channels are defined by 10MRs. The straight channels are approximately elliptical in shape, with a 5.3 Å×5.6 Å cross section, whereas the zigzag channels have a 5.1 Å×5.5 Å cross section.^50^ Because the cross sections are in the order of the kinetic diameters of isobutane and isobutene, researchers aim to separate mixtures of these compounds.

Fernandez et al. studied an MFI membrane prepared from silicate‐1.^51^ This framework is highly hydrophobic and stable up to 400 °C due to the high silicon/aluminum ratio.^50^ For single‐component loadings on the membrane at 363 K, the self‐diffusion coefficient of butane (*D*_butane_) is three orders of magnitude larger than that of isobutane (*D*_isobutane_). In particular, at a loading of four molecules per unit cell, the values were *D*_butane_=6×10^−9^ m^2^ s^−1^ and *D*_isobutane_=2×10^−12^ m^2^ s^−1^. Matsufuji et al.^52^ and Vroon et al.^53^ reported similar values for single‐gas permeances through MFI membranes. However, Fernandez et al. reported that in an equimolar mixture of butane/isobutane the diffusion coefficient of butane was two orders of magnitude lower than that in the single‐component measurement, whereas isobutane diffused slightly faster.^51^ Configurational‐bias Monte Carlo simulations showed that the butane molecules could be located either along the straight channels or in the zigzag channels of the membrane. Isobutane was located preferentially at the intersections of the straight and zigzag channels of the MFI membrane. Thus, the intersections provide more space for isobutane and probably serve as traffic junctions. In the equimolar mixture, the transport of butane along the straight channels in the *y* direction is halted because isobutane blocks the intersections.

Caro and co‐workers developed and patented a ZSM‐5 membrane prepared from tetraethylorthosilicate (TEOS) instead of silicate‐1.^54^ It showed high fluxes for 1‐butene but reduced selectivity for 1‐butene over isobutene, only slightly compared with membranes prepared from other silica sources. This was attributed to the presence of ethanol in the synthesis batch (originating from TEOS hydrolysis). SEM studies on silicate‐1‐MFI membranes from synthesis batches with and without ethanol indicated that the crystal size of all MFI membranes was reduced with increasing alcohol concentration. Smaller crystals have larger intercrystalline grain boundaries, and additional narrow non‐zeolite pores may form in the intercrystalline boundaries of the ZSM‐5 membranes. These pores increase the 1‐butene permeance in mixtures of 1‐butene/ isobutene gases.^55^

**Figure 6:**
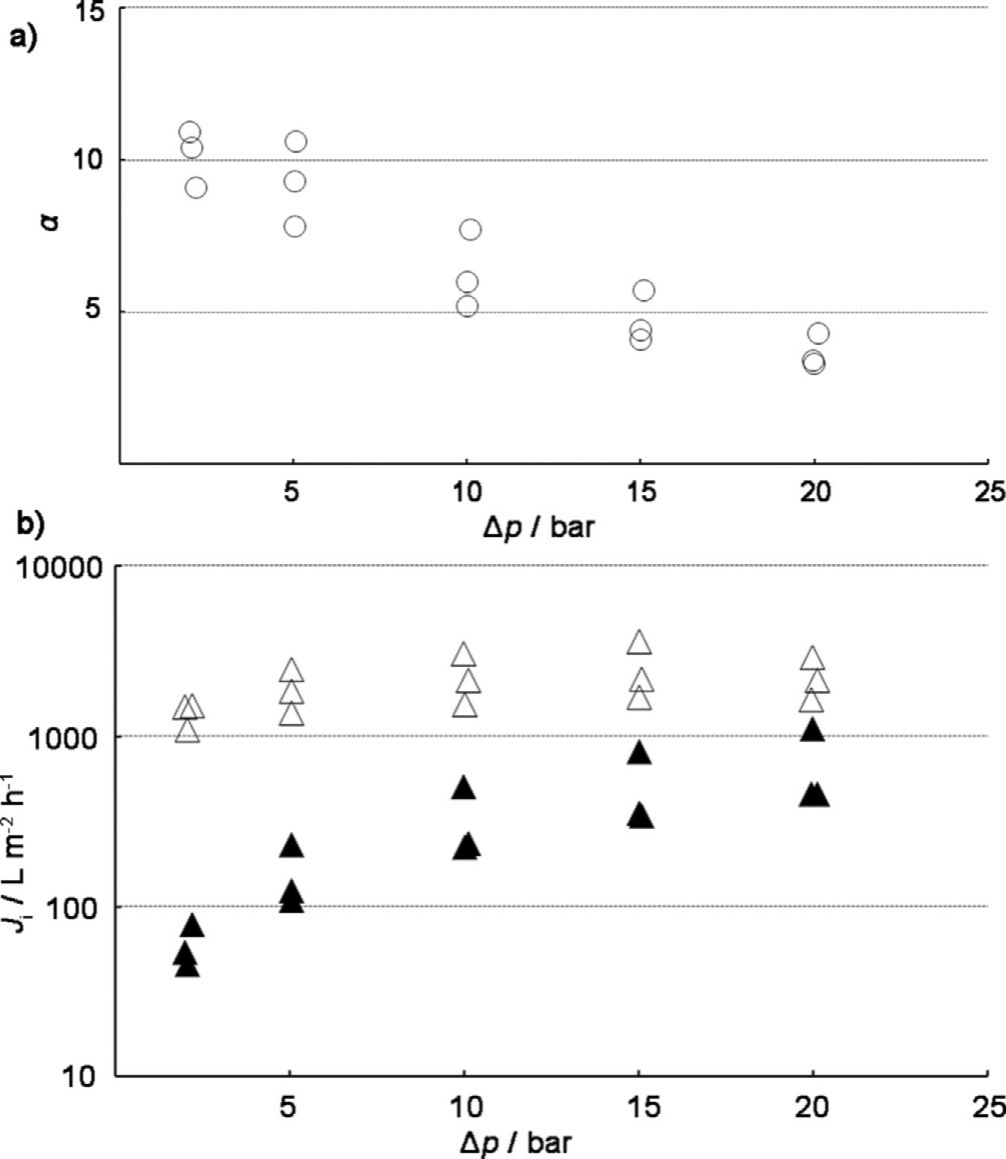
a) Decrease of the mixture separation factor, *α*, for an undiluted equimolar mixture of 1‐butene/isobutene through an MFI membrane at 403 K. b) Fluxes of 1‐butene (open symbols) and isobutene (filled symbols) from an equimolar mixture through an MFI membrane at 403 K. In both cases, the feed pressure was increased up to 21 bar, whereas the permeate pressure was constant at 1 bar. The three data points at each Δ*p* were derived from three independent membrane preparation and permeation tests.^56^

Voß et al. reported permeation tests by using an undiluted equimolar mixture of 1‐butene/isobutene at 403 K and an MFI membrane prepared from TEOS.^56^ Their studies showed that the mixture separation factor decreased from 10 to 5 when the pressure difference, Δ*p*, across the membrane increased from 1 to 20 bar. This significant Δ*p* is relevant to the practical operational pressure. The pressure of the equimolar undiluted feed was up to 21 bar and the permeate had a pressure of 1 bar. This drop in the separation factor impedes practical applications. The isobutene flux increases more steeply than that of 1‐butene (Figure 6), which causes a loss of selectivity with increasing pressure. Consequently, the 1‐butene to isobutene ratio in the permeate lessens with increasing Δ*p* and the selectivity for 1‐butene decreases. Chmelik et al. ran similar tests on butane/isobutane separation over MFI membranes prepared from silicate‐1, and reached similar conclusions.^57^

All of these examples used MFI‐type zeolites to separate butane from isobutane and 1‐butene from isobutene. Apart from the adsorption equilibrium of pure butane and 1‐butene, Wang et al. also studied the separation of their mixtures on ZSM‐5 zeolites.^15^ Adsorption isotherms were measured for pure and binary mixtures of 1‐butene and butane at 300 K and over a pressure range from 10^−4^ to 1 bar. The zeolites used were an all‐silicon ZSM‐5 and ZSM‐5 with Si/Al ratios of 120:1, 50:1, and 20:1, respectively (ion exchange was achieved with ammonium nitrate, setting protons as the nonframework cations). All four ZSM‐5 zeolites selectively adsorbed 1‐butene over butane. Moreover, the selectivity for 1‐butene increased at lower silicon/aluminum ratios. This can be explained by the presence of more available sites in zeolites with small silicon/aluminum ratios.^15^

Most experiments for C_4_ separation use 8MR zeolites. The pore sizes of these zeolites are smaller than those of the FAU‐ and MFI‐type zeolites, and match more closely with the kinetic diameters of C_4_ isomers. Here, we discuss C_4_ isomer separation by using SAPO‐17, DD3R, Si‐(CHA), ITQ‐32, and RUB‐41 zeolites. With the exception of SAPO‐17, these zeolites have high silicon/aluminum ratios and are therefore hydrophobic. SAPO‐17 is made by substituting framework atoms (P, Al) in AlPO_4_‐17 for silicon. This leads to the formation of one Brønsted center per unit cell.^58^ The 8MR channel system of SAPO‐17 has elliptical pore apertures with sizes of 3.6×5.1 Å. Richter et al. performed single‐component adsorption experiments (5 % of each C_4_ isomer in H_2_) and showed that *trans*‐2‐butene was selectively adsorbed on SAPO‐17 at low temperatures.^19^ In contrast, AlPO_4_‐17 has nearly the same adsorption capacity for all three butene isomers. It was concluded that SAPO‐17 was different because of the presence of silicon and, hence, the modification of lattice properties associated with the presence of Brønsted acid sites.^59^ Discriminating 1‐butene and *cis*‐2‐butene by SAPO‐17 is the result of differentiated electrostatic interactions between the negatively charged anion lattice and butenes with different polarity. *trans*‐2‐Butene has no permanent dipole moment, unlike *cis*‐2‐butene and 1‐butene. The orientation of 1‐butene and *cis*‐2‐butene dipoles towards the electrostatic field at the pore entrance is unfavorable for entering the pores, whereas *trans*‐2‐butene can enter the pore system easily.^19^

**Figure 7:**
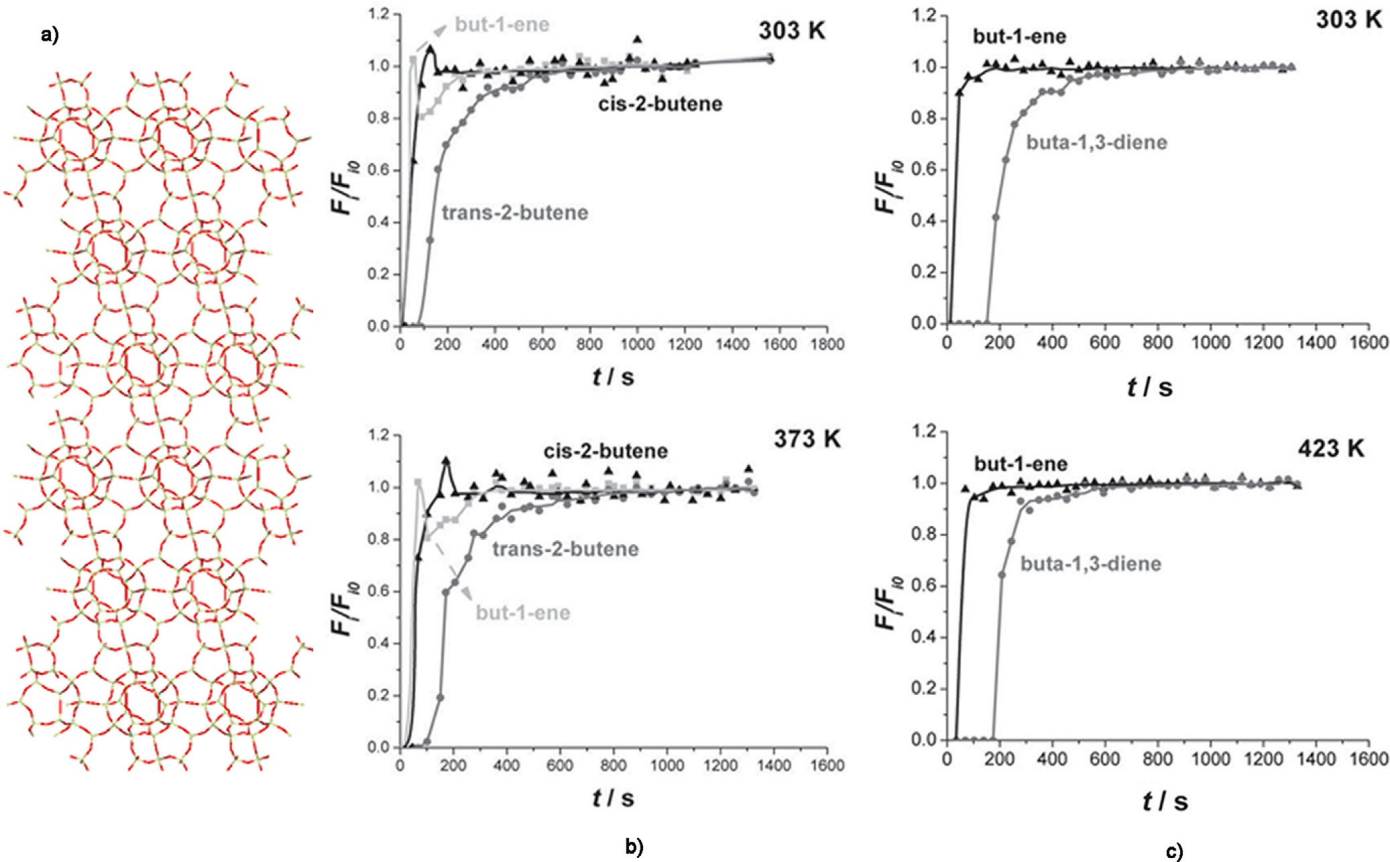
a) Building units and framework of the DD3R. Only the [4^3^5^12^6^1^8^3^] cages are accessible to smaller molecules, such as the C_4_ isomers.^17^ b) Normalized molar flows as a function of time for 1‐butene, *trans*‐2‐butene, and *cis*‐2‐butene in a ternary mixture (1:1:1) at 1.2 bar and 303 and 373 K. c) Normalized molar flows as a function of time of a 1:1 binary mixture of 1,3‐butadiene and 1‐butene at 1.2 bar and 303 and 423 K.^14^ Parts (b) and (c) reproduced with permission from The American Chemical Society.

Zhu et al.,^17^ Gücüyener et al.,^14^ and Jansen and coworkers.^25^ ran single‐component breakthrough experiments, multicomponent experiments, and performed molecular modeling studies with 1‐butene, *cis*‐2‐butene, *trans*‐2‐butene, and 1,3‐butadiene by using the hydrophilic decadodecasil 3R (DD3R) as adsorbent. The DD3R structure is stable at high temperatures and formed by three different types of cages. A unit cell of DD3R consists of six 10‐hedron [435661] cages, nine 12‐hedron [512] cages, and six 19‐hedron [435126183] cages. The 2 D pore topology of DD3R is constructed by linking the 10‐ and 12‐hedron cages through common faces. This yields 19‐hedron cages with a free volume of about 350 Å^3^. The latter are the only cages that are accessible for guest molecules. Each [435126183] cage is interconnected to three neighboring [435126183] cages through 8MR oxygen rings with a cross‐sectional diameter of about 4.5 Å.^17^, ^22^ The 8MRs are (at least in theory) wide enough for some C_4_ isomers.^22^ Figure 7 a shows the different cages and the framework of DD3R.^17^ The breakthrough measurements showed that, at temperatures between 303 and 373 K at 1 bar, DD3R is accessible to *trans*‐2‐butene and 1,3‐butadiene. 1‐Butene and *cis*‐2‐butene are excluded from the framework (see Figure 7 b,c).^17^, ^18^ This was explained through the critical diameter of the adsorptive molecules. The critical diameters of *trans*‐2‐butene (4.31 Å) and 1,3‐butadiene (4.31 Å) are slightly smaller than the free cross diameter of the 8MR and these molecules can enter into the cavities. However, the critical diameter of *cis*‐2‐butene (4.94 Å) is larger than the window size and the critical diameter of 1‐butene (4.46 Å) is comparable to it.^17^ Molecular modeling studies support this theory.^17^ When the 8MRs are indeed the smallest passage, the energy barrier of diffusion is simply the difference between the energy of component *i* in the cage, *E*_*i*cage_, and in the ring, *E*_*i*ring_. The permeabilities of the C_4_ isomers calculated from these barriers were in the order of *trans*‐1,3‐butadiene>*trans*2‐butene>*cis*‐1,3‐butadiene>*cis*‐2‐butene>1‐butene, which was consistent with the order reported by Zhu et al.^17^ and Gücüyener et al.^14^

Performing both breakthrough and single‐component adsorption experiments, Casty et al.^60^ and Palomino et al.^61^ showed that all‐silica 8MR zeolites Si‐(CHA) and ITQ‐32 had similar adsorption behavior to that of SAPO‐17 and DD3R. Pure silica CHA and ITQ‐32 adsorbed *trans*‐2‐butene quickly at temperatures of 273 and 298 K and pressures of 2 and 0.3 bar. These zeolites showed little or no adsorption for *cis*‐2‐butene and 1‐butene, even after an hour of contact time. The Si‐(CHA) consists of an 8MR channel system with window sizes of 3.50 Å×4.17 Å, whereas ITQ‐32 consists of interconnected 8MR and 12MR channels.^62^ The 8MR channels have window sizes of 3.5 Å×4.5 Å, whereas the 12MR channels have a diameter of 6.3 Å. Similar adsorption behavior of Si‐(CHA) and ITQ‐32 indicates that in ITQ‐32 the 8MR windows are the limiting factor for C_4_ diffusion.

**Figure 8:**
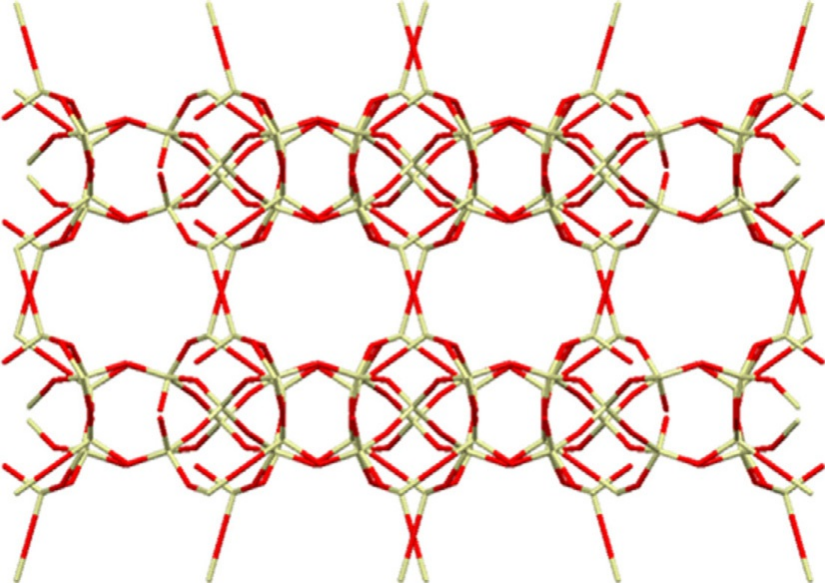
Skeletal model of the structure of RUB‐41, showing a projection along the 8MR channels.^15^

All of these zeolites prefer *trans*‐2‐butene over 1‐butene and *cis*‐2‐butene, owing to its smaller kinetic diameter (4.31 vs. 4.94 Å). These studies, however, were performed in the gas phase.^14^ Liquid‐phase adsorption studies were reported only for channels (4.0 Å×6.5 Å).^14^, ^15^ Figure 8 shows the skeletal model of the RUB‐41 structure, highlighting the projection along the 8MR channels. Wang et al. ran single‐component adsorption isotherms of isobutane, 1‐butene, and *trans*‐2‐butene on RUB‐41 up to a pressure of about 0.8 bar.^15^ Their results were consistent with the previous results on other 8MR zeolites: *trans*‐2‐butene adsorbed preferentially to isobutane.

**Figure 9:**
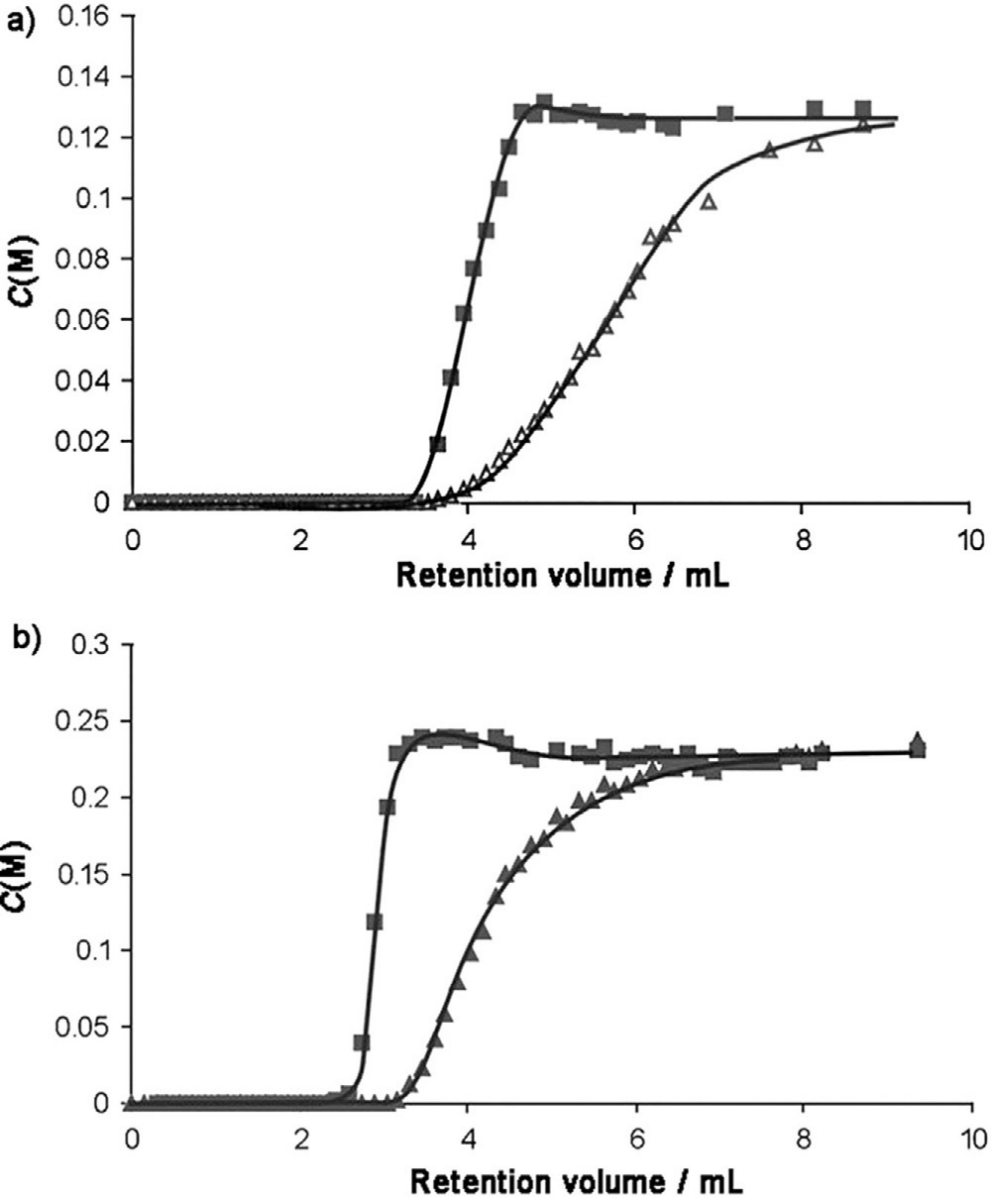
Breakthrough experiments with binary solutions of butene in cyclohexane on a 7.5 cm column filled with RUB‐41 at 298 K. *C*(M) denotes the effluent concentrations of a) a mixture of *cis*‐2‐butene (▴)/1‐butene (□), and (b) a mixture of *trans*‐2‐butene (▴)/1‐butene (□) as a function of eluted volume.^7^ Reproduced with permission from The Royal Society of Chemistry.

Tijsebaert et al. performed single‐component adsorption isotherms on *trans*‐2‐butene, *cis*‐2‐butene, 1‐butene, and isobutene in cyclohexane.^7^ The results indicated that *trans*‐2‐butene and *cis*‐2‐butene were much more strongly adsorbed than 1‐butene or isobutene. RUB‐41 zeolite prefers, therefore, both *trans*‐2‐butene and *cis*‐2‐butene over 1‐butene. This contrasts the order observed for other 8MR zeolites. Because the adsorption isotherms for RUB‐41 were recorded after sufficiently long times, it was concluded that the performance of RUB‐41 for 2‐butenes over 1‐butene was due to thermodynamic rather than kinetic effects. It may be that the 2‐butenes are more efficiently packed inside the pores than 1‐butene, or that 1‐butene might lose more of its conformational entropy in the pores. Breakthrough experiments with two binary butene mixtures were also performed. The first mixture consisted of *cis*‐2‐butene and 1‐butene and the second mixture consisted of *trans*‐2‐butene and 1‐butene. In both cases, 1‐butene eluted first (Figure 9). These results confirm that the separation can be performed in the liquid phase using RUB‐41.

## 5 Separating C_4_ Hydrocarbons with MOFs

Compared with zeolites, fewer MOFs were studied for the separation of C_4_ isomers. Pan et al. studied the adsorption properties of methanol, propane, propene, *n*‐butane, 2‐methylpropane, *n*‐pentane, 3‐methylbutane, *n*‐hexane, and 3‐methylpentane at 298 K by using [Cu(hfipbb)(H_2_hfipbb)_0.5_] (H_2_hfipbb=4,4′‐(hexafluoroisopropylidene)bis(benzoic acid)).^63^ The 2 D network of this hydrophobic MOF consists of microchannels that taper at 7.3 Å intervals, forming small cages (the tapering end diameter is ca. 3.2 Å). Interestingly, butane, propane, propene, and methanol were adsorbed in decreasing order, whereas the remaining compounds were not adsorbed. This shows that this MOF can separate normal C_2_, C_3_, and C_4_, from branched alkanes and normal hydrocarbons above C_4_.^64^ Molecular simulations also showed that about 40 % more *cis*‐2‐butene than *trans*‐2‐butene would fit into the cages. Notably, these findings support the cage size and shape effects, but not passage through the narrow neck region.^63^ Experimental studies are needed to understand the adsorption mechanism.

**Figure 10:**
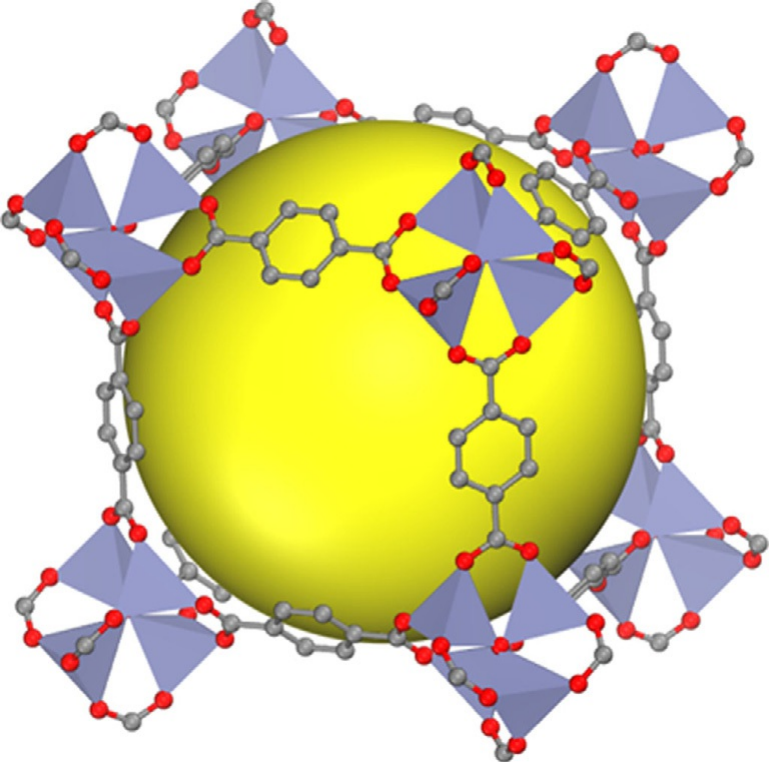
MOF‐5 consists of Zn_4_O units connected by linear 1,4‐benzenedicarboxylate to form a cubic network. Eight clusters (only seven are shown) constitute a unit cell and enclose a large cavity, as indicated by a yellow sphere with a diameter of 18.5 Å.

Li et al. designed MOF‐5, the first rigid MOF that showed permanent porosity after being fully desolvated or heated up to 573 K.^65^ It consists of Zn_4_O units connected by linear 1,4‐benzenedicarboxylate struts to form a cubic network.^66^ One unit cell of the framework consists of eight ZnO_4_ clusters (Figure 10) and encloses a large cavity with a diameter of 18.5 Å. Mertens and co‐workers prepared a MOF‐5‐CSA‐coated column to study its potential application in the separation of commercial natural gas and butane gas components.^64^ This MOF‐5‐CSA‐coated column was compared with a commonly used commercial column, Agilent HP PLOT S. Tests were run by using a natural gas sample that consisted mainly of C_1_–C_4_ alkanes (methane 97.1 %, ethane 1.7 %, propane 0.7 %, isobutene 0.2 %, and butane 0.3 %).^64^ The individual components were clearly baseline separated by both columns. However, the MOF‐5‐CSA‐coated column separated all five components more rapidly and without any other performance loss (0.8 min total separation time). The commercial column required 0.13 min longer and the distribution of peaks was nonuniform. To demonstrate the separation power of the MOF‐5‐CSA column, additional C_4_ components were added to the natural gas samples.^64^ The results showed that even different butene isomers were easily baseline separated, despite their similar vapor pressures and small amounts. The minimal separation time for all of the components was <4 min.^64^ The MOF‐5‐CSA column was operated over a period of five months, performing more than 300 chromatographic separations (in the range 40–50 °C) without any discernable loss of separation power.^64^ Further studies are required to test whether the MOF‐5 column maintains its selectivity when used for larger scale separations.

**Figure 11:**
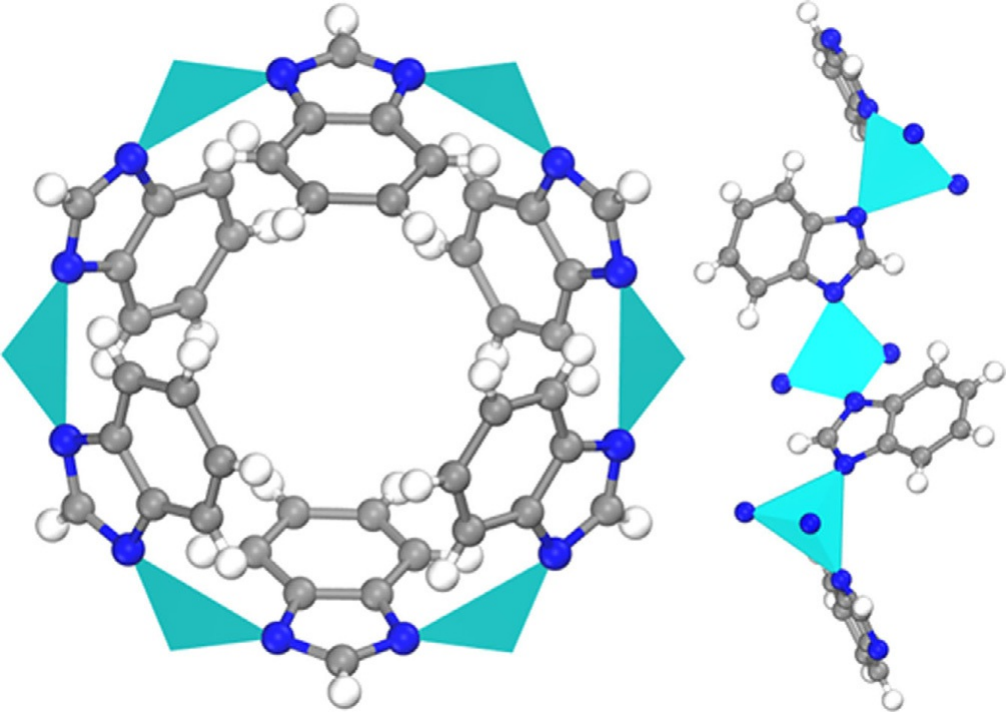
The main cavity entrance of ZIF‐7 (left), together with lateral (top right) and front (bottom right) views of one of the 6MR pore openings. ZnN_4_ clusters are represented as polyhedra.

ZIF‐7 belongs to the zeolite imidazolate frameworks (ZIFs) group of compounds, a subfamily of MOFs named after the resemblance of the metal–imidazolate–metal bond angles with that of the Si−O−Si angles of zeolites.^67^ This flexible MOF is formed by Zn^2+^ metal‐ion clusters linked through benzimidazole (BIM). It has six‐membered‐ring (6MR) pore openings with, in the optimized structure in vacuum, a diameter of 3 Å (see Figure 11 for details).

**Figure 12:**
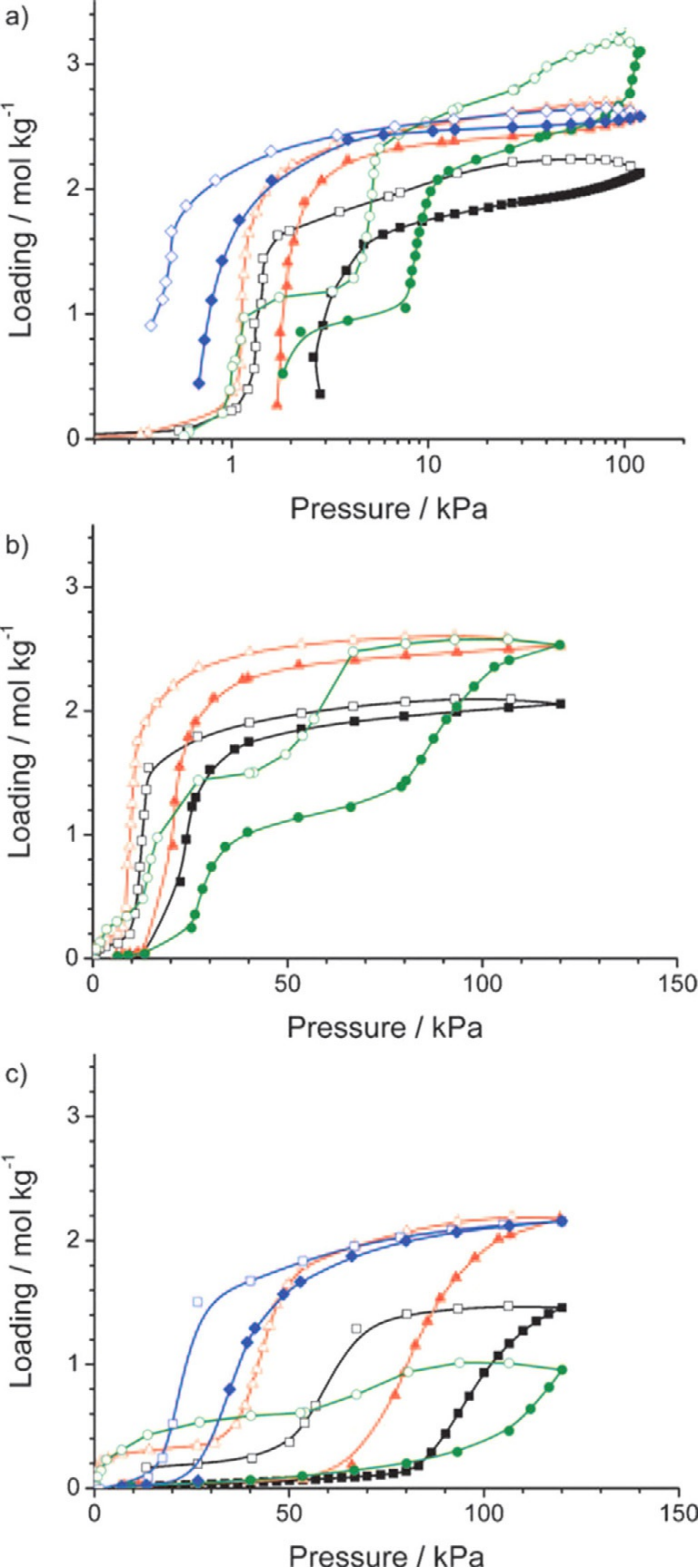
The adsorption isotherms of butane (blue), 1‐butene (black), *cis*‐2‐butene (red), and *trans*‐2‐butene (green) on ZIF‐7 at 298 (a), 338 (b), and 373 K (c). Closed symbols denote adsorption and open symbols denote desorption.^33^

Gascon and co‐workers measured single‐component adsorption isotherms of butane, 1‐butene, *cis*‐2‐butene, and *trans*‐2‐butene at 298, 338, and 373 K on ZIF‐7 (see Figure 12).^33^ All isotherms showed the typical characteristics of a flexible host material, including distinct steps and hysteresis in the adsorption and desorption branches. At 298 K and 1 bar, the MOF showed a higher saturation adsorption capacity for *trans*‐2‐butene over butane and *cis*‐2‐butene over 1‐butene.^33^ At 338 K, ZIF‐7 showed 25 % higher saturation adsorption capacity for *trans*‐2‐butene and *cis*‐2‐butene compared with that of 1‐butene.^33^

At a higher temperature, about 373 K, ZIF‐7 showed a higher saturation adsorption capacity for *cis*‐2‐butene and butane over 1‐butene and *trans*‐2‐butene.^33^ The main conclusion is that the separation of the C_4_ isomers on ZIF‐7 is temperature dependent, which enables the separation of the isomers studied.

Nair and co‐workers used two types of linkers to fine‐tune the pore size, hydrophilicity, and organophilicity of ZIFs.^68^ They demonstrated this through adsorption and diffusion measurements of hydrocarbons, alcohols, and water by using mixed‐linker ZIF‐8_*x*_‐90_100−*x*_ materials with a large range of crystal sizes. Varying the mixed‐linker composition parameter (*x*) allows continuous control of *n*‐butane, isobutane, butanol, and isobutanol diffusivities over two to three orders of magnitude. It also allows control of water and alcohol adsorption, especially at low activities.

**Figure 13:**
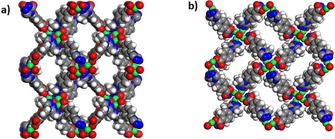
Space‐filling projections of [Cu_4_(μ_4_‐O)(μ_2_‐OH)_2_(Me_2_trz‐pba)_4_] with 3.5 Å×8.5 Å windows (a) and 4.5 Å×5.5 Å (b) windows in the *c* direction.

Another type of flexible MOF is Cu_4_(μ_4_‐O)(μ_2_‐OH)_2_(Me_2_trz‐pba)_4_ (Me_2_trz‐pba=4‐(3,5‐dimethyl‐4*H*‐1,2,4‐triazol‐4‐yl)benzoate).^31^ Its crystal structure has a three‐dimensional pore topology with two different windows and an estimated porosity of 57 %. The porous structure contains windows of 4.5 Å×5.5 Å (Figure 13 a) along *a* and *b* axes and 3.5 Å×8.5 Å pores along the *c* axis (see Figure 13 b). Lange et al. measured the single‐component isotherms of butane, isobutene, 1‐butene, and isobutene on [Cu_4_(μ_4_‐O)(μ_2_‐OH)_2_(Me_2_trz‐pba)_4_].^31^ The measurements were performed between 283 and 343 K at pressures up to 3 bar. By comparing the sorption isotherms of the studied C_4_ hydrocarbons, the first gate opening is observed at lower pressures.^31^ At higher pressures, only 1‐butene shows an additional sharp increase in loading. This indicates complete crystal‐to‐crystal transformation, whereas the adsorption of isobutane, isobutene, and *n*‐butane only leads to a partial transformation.^31^ However, the selectivity for 1‐butene is poor.

**Figure 14:**
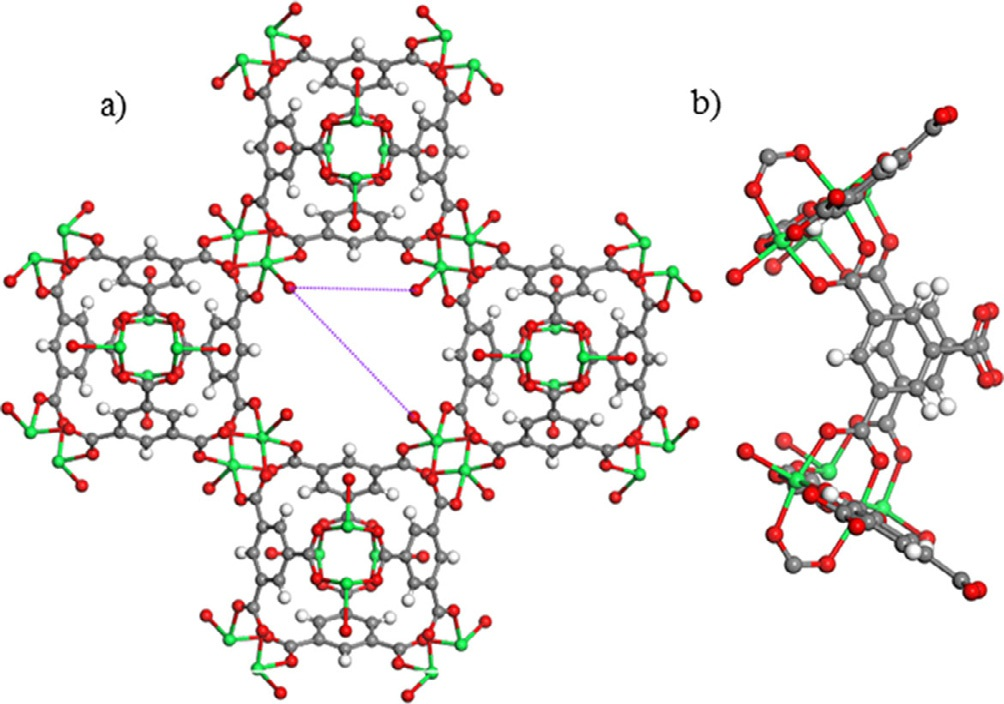
Pore window structure of [Cu_3_(BTC)_2_] (red=O; cyan=Cu; white=H; green=free Lewis acid site on Cu atom). a) Front view of a pore window (distances measured between the different ligation sites in the window are indicated in purple, and b) side view of a pore window.

Cu_3_(BTC)_2_ (BTC=1,3,5,‐benzene‐tricarboxylate), also known as HKUST‐1, is a Lewis acid MOF that has been studied extensively.^69^, ^70^ The main structural feature of Cu_3_(BTC)_2_ is a Cu^2+^ dimer with a Cu^2+^−Cu^2+^ distance of 2.63 Å. Twelve carboxylate oxygen atoms from the two BTC ligands bind to the four coordination sites of each of the three Cu^2+^ ions. In addition to the carboxylate ligands, one water molecule points towards the center of the pore and is coordinated to the copper center. When the coordinated water molecules are removed in vacuum, accessible Cu^2+^ centers are created that can act as Lewis acid sites. These paddle wheel units form a facecentered crystal lattice that possesses a three‐dimensional channel system with a bimodal pore size distribution.^71^ The larger pores are hydrophilic and have a diameter of about 9 Å, which define the 12 paddle wheel subunits that form a cuboctahedron. A smaller pore system of tetrahedron‐shaped side pockets, with a diameter of about 5 Å, are formed by four benzene rings (Figure 14).

The latter system is accessible from the large pores through windows with a diameter of about 3.5 Å.^20^

**Figure 15:**
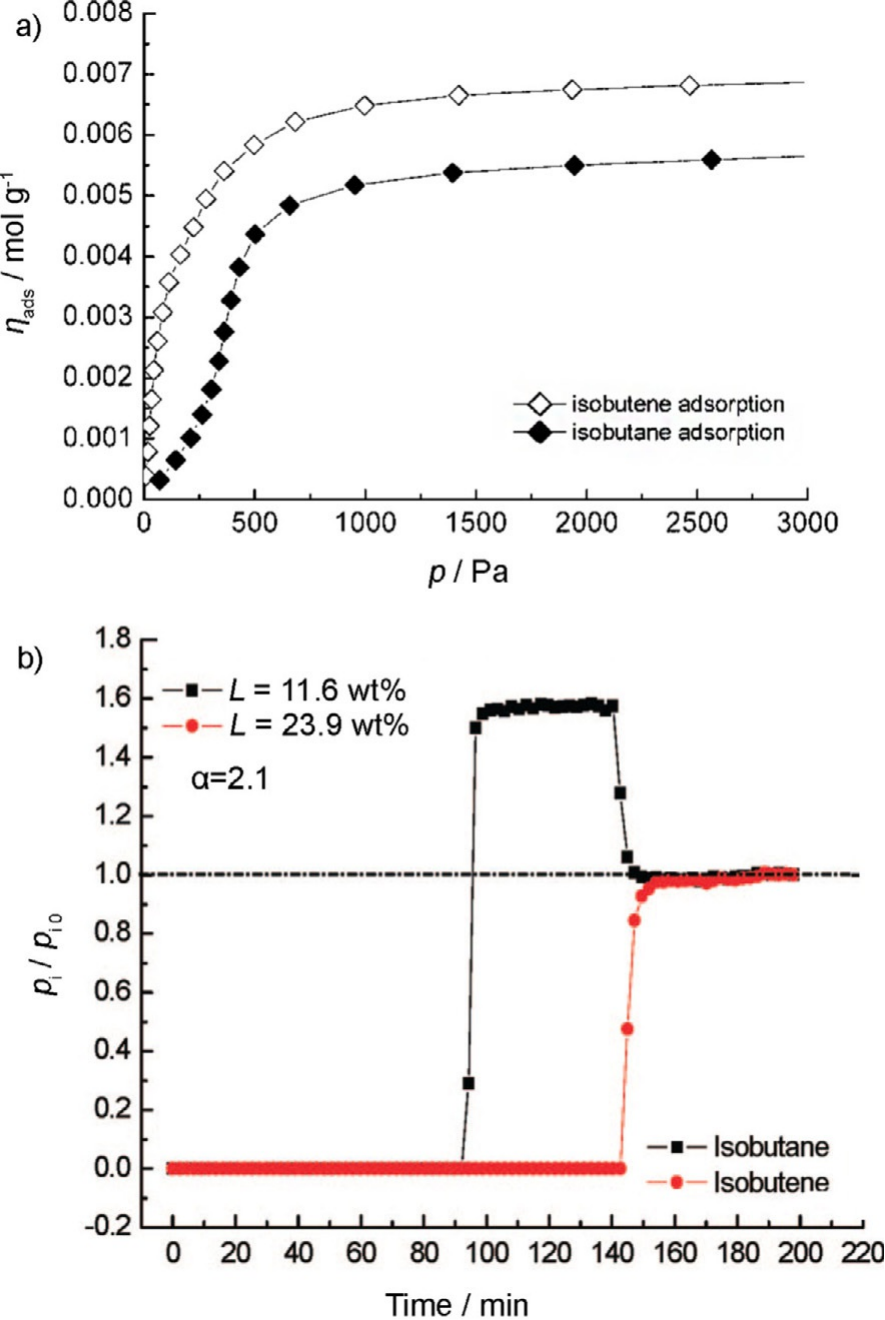
a) Comparison of single‐component isobutene and isobutane isotherms at 303 K on Cu_3_(BTC)_2_. b) Breakthrough curves for the separation of a mixture of isobutene/isobutene over Cu_3_(BTC)_2_ at 303 K.^20^ Reproduced with permission from The American Chemical Society.

Hartman et al. measured single‐component adsorption isotherms of isobutene and isobutane at different temperatures with Cu_3_(BTC)_2_ as an adsorbant.^20^ Figure 15 a shows the high‐resolution isobutane and isobutene adsorption isotherms at 303 K. Figure 15 b displays the breakthrough curves for the separation at the same temperature. Initially, isobutene and isobutene are completely removed from the feed stream. After about 90 min on stream, first isobutene breaks through and the partial pressure at the adsorber outlet rises to *p*_*i*sobutane_/*p*_isobutene_=1.6. After about 140 min on stream, isobutene breaks through and the adsorber inlet concentration is reached after about 150 min. This behavior is explained in terms of overshooting through the partial displacement of isobutane by isobutene from the adsorption sites in Cu_3_(BTC)_2_. The adsorption enthalpy of isobutene is only 5 kJ mol^−1^ higher than that of isobutene.^20^ This suggests that there are no strong π interactions between isobutene and the copper site. Instead, van der Waals interactions dominate.^72^

Alaerts et al. studied the separation of binary equimolar mixtures of *cis*‐butene and 1‐butene, *cis*‐butene and *trans*‐butene, and 1‐butene and *trans*‐butene on Cu_3_(BTC)_2_ in liquid hexane.^21^ The separation factors for these mixtures are 1.8, 1.9, and 2.4, respectively. These values indicate the preference of Cu_3_(BTC)_2_ for *cis*‐2‐butene over 1‐butene and *trans*‐2‐butene. Total uptakes varied from 12 to 21 wt %. The high preference of Cu_3_(BTC)_2_ for butenes in hexane reflects strong competition from the aliphatic solvent, with adsorption driven by π interactions with the Lewis acid sites. This is remarkable when compared with the findings of Hartmann et al.,^20^ who found that the gas‐phase separation of isobutane from isobutene was driven by van der Waals interactions. Notably, Alaerts et al. ran their experiments in the liquid phase, and therefore, at a higher pressure.^21^

**Figure 16:**
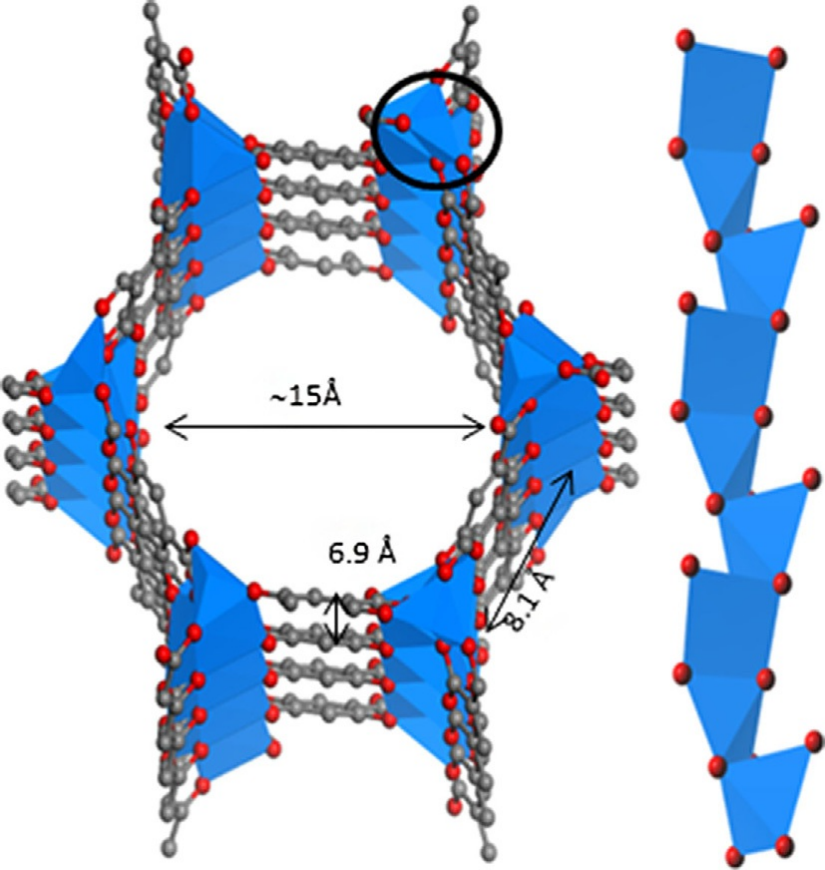
Left: Local structure of Fe‐MOF‐74; right: magnified view of the 1 D chain.^7^

M‐MOF‐74, also known as M‐CPO‐27 or M_2_(dobdc) (M=transition‐metal ion; dobdc=2,5‐dioxido‐1,4‐benzene dicarboxylate) also has coordinatively unsaturated metal sites.^73^, ^74^ Fe‐MOF‐74 has a honeycomb structure with open metal sites towards large pores of about 15 Å in diameter (Figure 16).^75^ All Fe^2+^ ions within this structure are coordinatively unsaturated, and the distance between them varies from 7 to 8 Å in both lateral and vertical directions. Such a large pore volume could accommodate relatively large molecules, such as C_4_ alkenes.

Kim et al. reported a DFT study showing that the Fe‐MOF‐74 structure was a promising candidate for 1‐butene separation.^76^ Binding energy calculations showed that 1‐butene bound preferentially over isobutene, *cis*‐2‐butene, and *trans*‐2‐butene.^76^ Particularly, 1‐butene had a 13–24 kJ mol^−1^ higher binding energy than those of the other isomers, which indicated that selective adsorption of 1‐butene on the MOF should be feasible. Experimental proof is needed because blocking or cooperative effects of other components in a mixture could lead to different results. Theoretical calculations indicated that 1‐butene could approach the metal binding sites more closely than the other butene isomers, enabling stronger bonding and π‐back‐bonding interactions between 1‐butene and MOF‐74.^76^ Potential π complexation is significantly hindered sterically for 2‐butenes and the adsorption depends largely on van der Waals interactions.^76^

## 6 Summary and Outlook

The number of studies published on C_4_ separation by using microporous materials is relatively small. Most of these studies focus on single‐component gas adsorption experiments. These experiments alone are insufficient for assessing the separation of mixtures of C_4_ isomers because blocking and cooperative effects in mixtures may lead to different behavior.^13^ Most of the experiments were run in the gas phase, at pressures up to about 1 bar, whereas industry usually uses higher pressures to minimize process costs. Ideally, separation should be studied in the liquid phase. There is a clear need for developing laboratory equipment that will enable in operando adsorption separation studies.

Regarding zeolites, an industrial C_4_ separation is unlikely with larger pore FAU zeolites and MFI membranes. In the Na‐FAU frameworks, all isomers can enter the large cages and specific interactions between the Na^+^ cations and the frameworks are too weak to effectively separate the isomers.^23^, ^24^, ^37^, ^40^ The separation process is mainly related to differences in the dipole moments and electrical polarizabilities of the C_4_ components. A low silicon/aluminum ratio provides more interaction sites and 1‐butene and 2‐butene are adsorbed preferentially. The adsorption of these molecules decreases considerably for zeolites with a high silicon/aluminum ratio.

MFI‐type materials have medium size pores and are hydrophobic due to the high silicon/aluminum ratio. They were studied for butane and isobutane separation, which is a diffusion‐controlled process. However, the selectivity of MFI‐type materials is still too low at practically relevant pressures. This is because the tunnel intersections of MFI membranes are blocked by the larger *iso* isomer, which halts linear isomers.^57^ Conversely, separation with 8MR zeolites may be feasible because their pore sizes are in the order of the kinetic diameters of the isomers. These zeolites (except for RUB‐41) adsorb *trans*‐2‐butene preferably over 1‐butene and *cis*‐2‐butene, in accordance with the critical diameter values.^14^ Additionally, RUB‐41 can separate 1‐butene from the 2‐butene isomers due to thermodynamic effects.^7^ That said, the selectivities of the 8MR zeolites are still too low to meet high‐purity olefin demands, even after many adsorption/desorption cycles.

MOFs are attracting increasing attention because their flexibility and unsaturated metal centers might provide increased selectivity for gas separation.^2^ Indeed, a column coated with MOF‐5 showed remarkable performance by baseline separating all butene isomers.^62^ The results reported for the flexible ZIF‐7 framework are also promising. The framework shows temperature‐dependent preferences for different C_4_ isomers.^32^ However, only single‐component isotherms over a low‐pressure range were reported. Breakthrough experiments at higher practically relevant pressures are needed because slight changes in external stimuli can cause considerable changes in the framework and degree of flexibility. Moreover, the flexibility of MOFs could be problematic in an industrial application because the framework has to face permanent stress through heating, outgassing, and cooling during adsorption.^31^, ^72^ Concerning Lewis acidic MOFs, the liquid‐phase separation in aliphatic solvents is an attractive option because in the presence of an aliphatic solvent butenes are preferably adsorbed.^21^

An attractive, but much less explored, method for C_4_ separations is separation through π complexation. The advantage of this method arises from strong interactions being established between adsorbent and adsorbate, which are stronger than those involving only van der Waals interactions. Such strong interactions allow higher selectivity and adsorption capacity to be achieved. Importantly, the bonds formed by π complexation are still weak enough to be broken by changing the parameters of a separation process, for example, pressure or temperature. Therefore, separation by π complexation could be a simple and efficient process, particularly by PSA or TSA. Indeed, some studies were reported on Ag^+^‐ or Cu^+^‐modified zeolites.^60^ So far, the most promising results were obtained for Cu‐Y frameworks, but one must bear in mind that this better performance comes at a price.^42^ One ton of sodium costs about $2000, whereas a ton of copper costs about $6000 (2015 prices). The use of copper instead of sodium would increase the adsorbent costs. Nevertheless, this might be compensated for by the fact that separations by PSA, especially in the gas phase, are relatively simple and highly efficient in terms of product purity and recovery.

This review illustrates that most studies on C_4_ separations have mainly focused on zeolites, whereas the potential of MOFs is largely unexplored. We believe that MOFs, with their unique tailored functionalities, hold the key to sustainable C_4_ separation processes in the coming decade. We foresee that separation by π complexation with MOFs will offer advantages for adsorptive separations due to the possibility of tuning the interactions established between adsorbent and adsorbate. Furthermore, experimental studies should also focus on both low‐ and high‐pressure separations. Low‐pressure separations are enthalpy driven, but at higher loadings the separations become entropic in nature, when molecules are highly structural ordered. Consequently, entropy‐driven separations would allow for separations by using high loading, and hence, increase efficiency.

## Conflict of interest

*The authors declare no conflict of interest*.

## Biographical Information

Mascha Gehre received a B.Sc. in chemistry (2013) and an M.Sc. in photonics and molecular simulations (2015) from the University of Amsterdam in the Netherlands. Her current placement is with a FinTech Institution in the Netherlands. She is interested in software development, big data analysis, and computational chemistry.

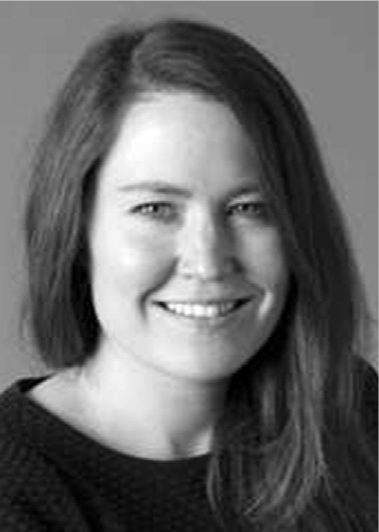

## Biographical Information

Zhiyong Guo received his Ph.D. from Changchun Institute of Applied Chemistry, Chinese Academy of Sciences, in 2010 under the supervision of Prof. Hongjie Zhang. Then, he worked as a postdoctoral researcher with Prof. Banglin Chen at the University of Texas, San Antonio (2010–2011); with Prof. Wenyu Huang at Iowa State University/Ames Laboratory (2011–2013); and with Prof. Sourav Saha at Florida State University (2014–2015). From 2015 to 2016, he was a postdoctoral researcher in sustainable chemistry with Dr. Stefania Tanase and Dr. Jarl Ivar van der Vlugt at the University of Amsterdam. Since 2016, he has been a professor at the College of Materials at Fuzhou University, P. R. China. His scientific interests are focused on the design, synthesis, and optoelectronic functions of organic porous frameworks.

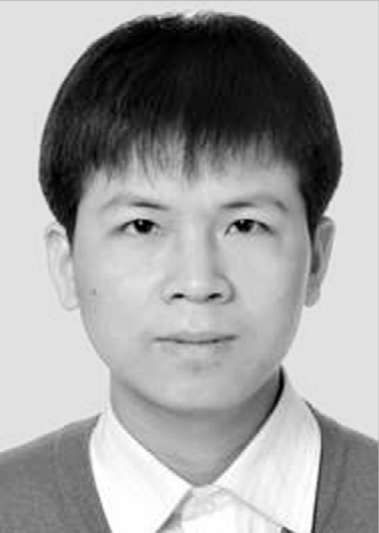

## Biographical Information

Gadi Rothenberg obtained his B.Sc. in chemistry (magna cum laude) from the Hebrew University of Jerusalem in Israel in 1993, and his Ph.D. in applied chemistry (summa cum laude) from the same university in 1999. After two years as a Marie Curie Fellow at the University of York, he settled at the University of Amsterdam. Since 2008 he has been Professor and Chair of Heterogeneous Catalysis and Sustainable Chemistry. He teaches courses on catalysis, thermodynamics, and scientific writing. He has published two books and over 180 papers in peer‐reviewed journals. His textbook *Catalysis: Concepts and Green Applications* is a Wiley‐VCH bestseller. He also has 15 patents and cofounded the companies Sorbisense A/S, Yellow Diesel BV, and Plantics BV. His latest inventions are a new catalyst for cleaning cyanide from wastewater, and a supercapacitor material made from carbon and nitrogen.

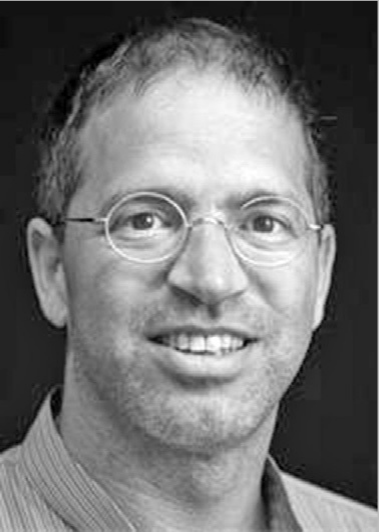

## Biographical Information

Stefania Tanase received her B.Sc. in chemistry and physics in 1996 from the University of Bucharest and her M.Sc. in advanced inorganic synthesis in 1997 from the Polytechnic University of Bucharest in Romania. She obtained her Ph.D. in inorganic chemistry in 2002 from the University of Bucharest, for which she received a Young Investigator Award. She was a postdoctoral (2001–2004) and independent VENI research fellow (2005–2008) at the University of Leiden in the Netherlands. In 2009, she joined the University of Amsterdam as a senior researcher and since 2011 as Assistant Professor in Heterogeneous Catalysis and Sustainable Chemistry, leading the Inorganic Materials group. Her research focuses on the development of synthetic methodologies for the preparation of inorganic and hybrid inorganic–organic materials with applications in molecular separations, magnetism, molecular sensing, catalysis, and as proton conductive membranes for fuel cells. She has coauthored over 75 publications and 2 book chapters.